# Obesity: an evolutionary context

**DOI:** 10.1093/lifemeta/loac002

**Published:** 2022-04-29

**Authors:** John R Speakman, Joel K Elmquist

**Affiliations:** Shenzhen Key Laboratory of Metabolic Health, Center for Energy Metabolism and Reproduction, Shenzhen Institutes of Advanced Technology, Chinese Academy of Sciences, Shenzhen, China; School of Biological Sciences, University of Aberdeen, Aberdeen, UK; State Key Laboratory of Molecular Developmental Biology, Institute of Genetics and Developmental biology, Chinese Academy of Sciences, Beijing, China; CAS Center of Excellence in Animal Evolution and Genetics, Kunming, China; Departments of Internal Medicine and Pharmacology, Center for Hypothalamic Research, University of Texas Southwestern, Dallas, TX, USA

**Keywords:** obesity, BMI, body fat, adiposity, evolution, selection, adaptive, leptin resistance, metabolic programming, dual-intervention point model, set-point model

## Abstract

People completely lacking body fat (lipodystrophy/lipoatrophy) and those with severe obesity both show profound metabolic and other health issues. Regulating levels of body fat somewhere between these limits would, therefore, appear to be adaptive. Two different models might be contemplated. More traditional is a set point (SP) where the levels are regulated around a fixed level. Alternatively, dual-intervention point (DIP) is a system that tolerates fairly wide variation but is activated when critically high or low levels are breached. The DIP system seems to fit our experience much better than an SP, and models suggest that it is more likely to have evolved. A DIP system may have evolved because of two contrasting selection pressures. At the lower end, we may have been selected to avoid low levels of fat as a buffer against starvation, to avoid disease-induced anorexia, and to support reproduction. At the upper end, we may have been selected to avoid excess storage because of the elevated risks of predation. This upper limit of control seems to have malfunctioned because some of us deposit large fat stores, with important negative health effects. Why has evolution not protected us against this problem? One possibility is that the protective system slowly fell apart due to random mutations after we dramatically reduced the risk of being predated during our evolutionary history. By chance, it fell apart more in some people than others, and these people are now unable to effectively manage their weight in the face of the modern food glut. To understand the evolutionary context of obesity, it is important to separate the adaptive reason for storing some fat (i.e. the lower intervention point), from the nonadaptive reason for storing lots of fat (a broken upper intervention point). The DIP model has several consequences, showing how we understand the obesity problem and what happens when we attempt to treat it.

## Background

Adipose tissue is a fundamentally important component of our body. Individuals who do not have any adipose tissue to store fat (lipodystrophy) have severe metabolic problems [1, 2]. Surgical implantation of adipose tissue to mice under a similar condition can reverse some of these negative impacts [3]. Having some adipose tissue and body fat is required for health and is, therefore, advantageous. It is also obvious that individuals who have excessive body fat are similarly metabolically compromised. For example, individuals with a body mass index (BMI) > 35 have 43 times (in males) and 93 times (in females) the risk of developing type 2 diabetes than individuals with a BMI of 22 [4, 5]. Somewhere between these extremes, there must be a level of body fat that is optimal, in the sense that it allows us to avoid the metabolic consequences of having too little or too much. There are several potential models for the nature of this regulation. We may have a system that regulates around a so-called set point (SP) or adipostat [6, 7]. Alternatively, we may have a system that prevents us from having too much or too little, but is relatively insensitive to changes across a wide range of intermediate values [8–10]. Whatever the system we have, it presumably evolved during our ancient past. This raises several questions: what were the selective pressures that influenced the evolution of the system? Assuming that we are correct in proposing the existence of such a control system for fatness, then something has apparently gone wrong with it because increasing numbers of people seem to have lost control of their fat levels. What has gone wrong? Finally, what are the other implications of this system for our attempts to reverse the obesity problem, for example, during exercise interventions? The aim of this paper was to discuss the evolutionary aspects of these three questions.

## Question 1: What is the nature of the regulation system?

**Figure 1:**
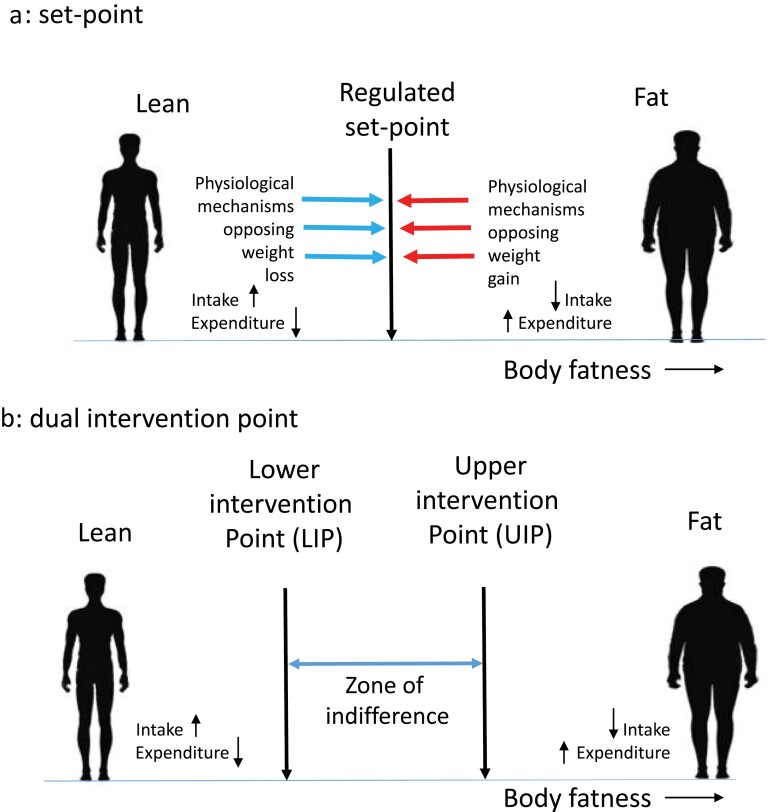
Two different models of body fat regulation. (a) SP system. Deviations of fatness above and below the regulated SP invoke strong physiological responses to resist the change. Elevations above the SP lead to reduced intake and increased expenditure, while the converse happens when fatness falls below the SP. (b) DIP system. Now there are two intervention points on the spectrum of body fatness: a lower point below which physiology resists further loss and an upper point above which physiology resists further gain. Between the two is a zone of indifference where weight is not physiologically regulated

Body fatness is a regulated trait that has significant heritability [11]. This implies that there are biological systems that regulate the amount of energy consumed and stored; this matches our personal experience. If you have ever been on a calorie-controlled diet, you will know that once you have lost a few kilograms of weight (fat) you develop powerful feelings of hunger, potentially leading you to break the diet, and once the diet has been broken you often return to your original weight. However, although less often performed, attempts to do the opposite (i.e. engage in rapid weight and fat gain) meet with similar opposing forces that stop weight accumulation and drive the body fatness back down again [12]. Kennedy [6] suggested that the reason for this pattern is because our bodies have an “adipostat” that controls the levels of our body fat around an SP, much like a central heating-aircon system controls the temperature of our houses (Fig. 1a). By this SP model, a signal from our bodies tells the brain how fat we are, and this is compared with the SP somewhere in the brain, and if the levels of fat are above the SP, we intervene by reducing our intake and/or elevating our expenditure, to bring the level of stored fat down again. Similarly, if the signal indicates that we have lower fat than the SP, we intervene in the opposite direction. Several hormones, including leptin and insulin, have the required properties to act as the adiposity signal [7, 13, 14], because their circulating levels reflect the levels of body fat [15].

These peripheral hormones interact with various neuroendocrine systems in the hypothalamus and brainstem that have been established to be involved in the regulation of both food intake and energy expenditure [13, 14, 16–18]. These circuits in the brain that control food intake and body weight might form the molecular basis of an SP control system. The discovery of these systems was catalyzed in large part by the identification of leptin and the cloning of the melanocortin receptors, and by the identification of the key role melanocortin neurons play in regulating food intake, body weight, and metabolism [15, 19–22]. The central melanocortin system is composed of pro-opiomelanocortin (POMC) neurons producing melanocortins such as α-melanocyte-stimulating hormone (α-MSH). POMC neurons are located in the arcuate nucleus and the nucleus of the solitary tract in the brainstem [23–25].

A distinct population of adjacent neurons in the arcuate nucleus expresses agouti-related peptide (AgRP) which blunts the actions of α-MSH to regulate food intake and glucose homeostasis. These melanocortin neurons are important targets of leptin as subsets of these neurons express leptin receptors (LEPRs) [24, 25]. They act on melanocortin 4 receptors (MC4Rs) and melanocortin 3 receptors (MC3Rs), which are widely expressed in the central nervous system [25–28]. A key point is that MC4Rs are expressed by neurons that regulate feeding and autonomic function where they control energy balance. Notably, human mutations in MC4Rs may represent the most common mutations causing monogenic obesity [29, 30]. The association of the melanocortin system to the regulation of body weight originated in the study of a mutant mouse (the agouti mouse) which had a bright orange coat color as well as severe obesity [31]. The two genes affecting coat pigmentation identified in mice were the melanocortin 1 receptor (MC1R) and agouti. Mutation of MC1R is the most common cause of red-colored hair in humans. The association between the orange coat color and obesity in the Agouti mouse led to speculation whether these traits might also have a molecular link in humans. Agouti is normally expressed only in the skin and is an antagonist of MC1R, blocking the production of melanin in favor of pheomelanin which has an orange color. Agouti mice have a mutation in the promoter region of the agouti gene that puts agouti under the transcriptional control of a ribonuclear protein Raly [32], which is universally expressed, resulting in widespread production of the agouti peptide, instead of its production being restricted to the skin. Expression of agouti peptide in the brain antagonizes MC4R and the mice become severely obese. However, this linkage of coat color to obesity depends only on this unusual expression pattern for the agouti peptide in this mutant mouse strain. Agouti-related peptide (AgRP) expressed in the hypothalamus is the endogenous antagonist of MC4R. Importantly, overexpression of AgRP that leads to obesity does not antagonize the MC1R and hence does not affect coat color. Hence, there is no molecular reason in humans to anticipate a general link of obesity to pigmentation effects—such as having red hair.

Abundant evidence has demonstrated that melanocortin neurons are reciprocally regulated by leptin. This includes regulating the acute activity (firing rate) as well as the transcriptional regulation of AgRP and POMC gene expression [18, 24, 33]. For example, the membrane potential of AgRP neurons is inhibited by leptin [34, 35], whereas the POMC neurons are stimulated [34]. Notably, neonatal deletion of AgRP neurons has minimal effects on feeding, but their ablation in adults causes starvation and death [36, 37]. Finally, a recent study demonstrated that adult-specific deletion of LEPRs has a very large effect to increase food intake and body weight [38]. Collectively, these studies establish the critical role of leptin signaling in AgRP neurons for the regulation of energy balance and glucose metabolism. Collectively, AgRP and POMC neurons can be viewed as key integratory nodes sensing the metabolic state ultimately leading to coordinated modulation of food intake and metabolic rate.

Another central target of leptin is neurons in the ventral medial nucleus of the hypothalamus (VMH). LEPRs are expressed in the dorsal medial VMH [39, 40]. The VMH has long been recognized as a crucial regulator of whole-body metabolism [41, 42]. Classic lesioning studies established a role for the VMH in regulating energy balance [43]. The VMH receives key metabolic signals regarding nutritional status and, in turn, regulates appetite, glucose, and lipid homeostasis including peripheral insulin action [18, 44]. VMH neurons express the orphan nuclear receptor steroidogenic factor 1 (*Nr5a1*). Notably, expression during development is required for the VMH to form [45–47]. VMH neurons also regulate adaptive responses to metabolic challenges, such as high-fat diet (HFD) and exercise [48–50]. In addition, activation of VMH neurons increases sympathetic nervous system activity to key metabolic tissues and can induce glucose uptake in the skeletal muscle, brown adipose tissue (BAT), and increase lipolysis in white adipose tissue (WAT) [51–54].

Neurons in the VMH integrate signals such as leptin, insulin, estrogen, and glucose to regulate energy expenditure and glucose homeostasis as well as respond to metabolic challenges such as HFD and exercise [18, 55]. For example, mice lacking LEPR only in SF-1 neurons of the VMH show increased weight gain, increased adiposity, and impaired glucose homeostasis [56, 57]. Additionally, mice lacking only in the VMH display increased body weight and adiposity, when challenged with an HFD, due to reduced energy expenditure. As SF-1 is a transcription factor, SF-1 probably regulates genetic programs in the VMH that link metabolic challenges to coordinated whole body responses. Clearly, the VMH and melanocortin neurons are not the only sites of leptin action. They are highlighted here because much future work needs to be done on these circuits. However, several excellent recent reviews have highlighted the roles of other brain regions in regulating feeding, body weight, and metabolism including brainstem circuits [16–18,58].

**Figure 2:**
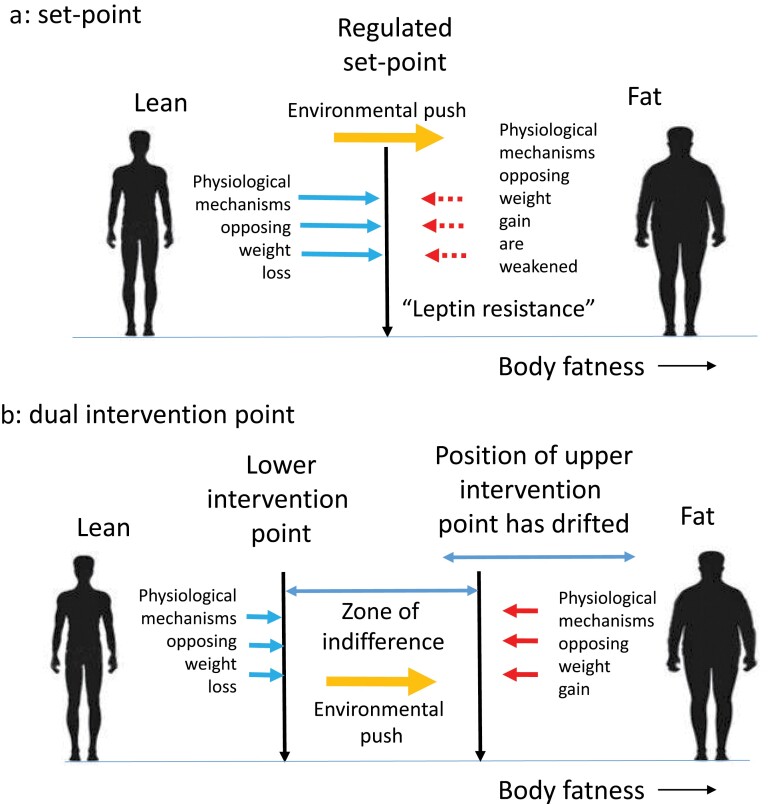
Two ideas why we become obese in modern society based on the models in Fig. 1. (a) Under the SP model, obesity develops because there is a weakening of the physiological regulatory response to increasing leptin levels, which has been termed “leptin resistance.” In the face of an environmental push, the SP is no longer able to resist weight gain. (b) Under the DIP model, it is suggested that the position of the upper intervention point has drifted in evolutionary time (the “drifty gene” hypothesis); hence, under an environmental push, individuals become obese to differing extents until they reach their own intervention points. The absence of a response to leptin at the LIP is not, therefore, seen as a pathological state of “leptin resistance” but part of how the system always works

Although our understanding of the food intake regulation system in the brain has expanded enormously, this molecular knowledge has provided few insights regarding the nature of the hypothesized “SP,” and how this is encoded in the brain. This might be because of several issues with an SP interpretation of the way the fatness control system works. The first is that the system appears to be asymmetric in how it responds to incoming adiposity signals [59, 60]. For example, lowered leptin seen following fasting evokes quite strong responses in terms of increasing feeding and reduced expenditure [44, 61]. At the limits, this is exemplified by the ob/ob and db/db mice [62, 63] that lack leptin and the long form of the LEPR, respectively, and have elevated food intake, suppressed metabolism, and enormous obesity [15]. Importantly, this low leptin signal appears strongly conserved between mice and humans as evidenced by individuals with loss-of-function mutations in the same genes [64]. One way to envision these responses is that mice or humans lacking leptin, or LEPRs, live in a constant state of perceived “starvation.” Notably, elevating (repleting) leptin levels leads to profound behavioral changes and weight loss in ob/ob mice [65–67] and leptin-deficient humans [68]. However, in humans and mice that are not leptin deficient, it results in only rather modest reductions in food intake, increased expenditure, and loss of body fat [69, 70]. To explain this asymmetrical response, researchers have proposed the concept of “leptin resistance” [59, 71, 72] (Fig. 2a). That is, the response to leptin was lower than expected, and so it was suggested that something must be stopping the leptin from working effectively—individuals were resistant to the leptin signal.

There are distinct mechanisms that influence the impact of rising leptin compared with falling leptin. However, calling these mechanisms “leptin resistance” may lead us to believe the failure to respond as leptin arises as a pathological response akin to “insulin resistance”—the pathological failure to respond to insulin. The ultimate extension of this viewpoint is to regard obesity as a pathological disease because of the failure to appropriately respond to leptin, like the insulin resistance that proceeds β-cell failure in type 2 diabetes, and call it “type 2 obesity” [73]. The key point, however, is that as a concept, pathological leptin resistance is only necessary if one believes in the first place that body fatness is controlled by an SP system. If it is not controlled by such a system, then the concept may be redundant. It is important that leptin has a myriad of effects that are mediated by multiple cell types and several central circuits. Leptin resistance is almost always discussed and defined as a failure of pharmacological doses of peripherally administered leptin to suppress food intake and body weight with the presence of diet-induced obesity. However, it is clear that not all actions of leptin become resistant to the endogenous leptin signal. For example, a lack of leptin action (either genetically or following fasting) inhibits pubertal development or leads to a complete shutdown of the reproductive axes in adults. However, in the context of obesity (with high leptin levels), the neuroendocrine effects of leptin on the reproductive axis often remain intact. This dual responsiveness may actually be adaptive because body fatness may provide an important energy reserve to support reproduction (see later). However, under certain conditions such as those seen in women with polycystic ovary syndrome, it is likely that leptin and insulin resistance contributes to the neuroendocrine and metabolic phenotype in these subjects.

The concept of leptin resistance grew out of the observation that there was a lack of responsiveness of obese individuals to leptin administration. However, the successful use of leptin for the treatment of individuals with lipodystrophy and leptin deficiency is impressive [74]. Collectively, this work has highlighted the distinct physiological roles of leptin to regulate glucose homeostasis, independent of the effects on food intake and body weight. Along these lines, reduction of bioactive leptin levels in the context of obesity induces leptin sensitization and improves leptin action [75]. Hence, we argue that we need to think about the totality of leptin action on neuronal and non-neuronal targets to understand the effects of low and high levels of leptin, and this may be incompatible with a simple “SP” theory and the concept of leptin resistance [75].

An alternative regulatory system to an SP system is known as the dual intervention point (or DIP) system [8–10] (Fig. 1b). In this model, there is not a single-regulated SP but rather two critical points—known as the lower (LIP) and upper intervention points (UIP). In this system, body fatness between the two points is not physiologically regulated and exhibits a “zone of indifference.” This is consistent with the observation that people in the USA often gain weight during the holiday period averaging about 0.3 to 0.5 kg, but this weight is subsequently not lost [76]. Moreover, many people gradually increase body weight year upon year [77] without any countervailing response to reduce it. If there was a strong SP in place, one might expect the holiday gain to be reversed once the holidays were over, and the slow year-on-year accumulation to be resisted. The advantage of the DIP model is that it combines an unregulated zone where environmental factors dominate weight change (such as during the holiday season and over multiple years) with the regulated parts of the SP model (such as the resistance to weight loss when dieting or over-feeding) into a combined hybrid system. An important aspect of the DIP model is that, at the LIP, the system marks a point that only generates responses to decreases in body fatness. This is completely consistent with the observed response to leptin in the fasted state [61]. When leptin falls, it is sensed by the brain as a threat of starvation and thus there is a strong opposing reaction. However, increases in leptin above this LIP provoke no response. In this model, there is no need to invoke the concept of pathological leptin resistance. The molecular mechanisms detailed above that result in the absence of a response to increasing adiposity at the LIP are an integral part of the DIP model. Under a DIP, the identified mechanisms described as pathological “leptin resistance” are the expected non-pathological mechanisms of asymmetrical leptin action at the LIP.

## Question 2: What were the key selective pressures that influenced the evolution of that system?

Whether one believes in an SP or a DIP system, it seems likely that the regulatory system evolved in response to specific selective pressures against storing too little and too much fat. What might be the problems with storing too little fat? One of the primary functions of adipose tissue is as a storage organ for lipids. Lipids not only are preferable for storage to other macronutrients, such as protein and carbohydrate because they have a high energy density (39 kJ/g) when compared with 17 kJ/g for carbs and protein, but also are anhydrous and do not need to be stored with water. For example, glycogen needs to be stored with 3 times its weight as water [78]. Hence, storing energy as lipids maximizes the energy stored per gram weight. If we do not have adipose tissue, then lipids need to be stored elsewhere—as occurs in lipodystrophy—and that leads to severe metabolic dysfunction (see references above). So, adipose tissue probably evolved as a specific tissue that can serve as a storage organ for fat, thereby avoiding the metabolic consequences of storing fat elsewhere [79]. In addition, the specific depots in which fat mass is expanded are also important. For example, abundant work has demonstrated that expansion of visceral fat depots has significant adverse metabolic effects. In contrast, expansion of subcutaneous depots can be protective against obesity-induced metabolic disturbances [80, 81]. This response is exemplified by mice transgenically expressing adiponectin in the context of total leptin deficiency (ob/ob mice) results in massive obesity, an expansion of the subcutaneous adipose depots, and correction of the metabolic derangements characteristic of total leptin deficiency [81].

The energy stored in fat can be used to make good shortfalls in the external energy supply. If the food supply fails for some reason, then stored fat can be used to sustain energy expenditure. Perhaps, the earliest idea about fat storage (and hence an evolutionary model of obesity) is that it evolved as a buffer against failures in the environmental food supply. This idea is commonly known as the Thrifty gene hypothesis or TGH [82] although Neel was actually attempting to explain the evolution of insulin resistance and diabetes (which he suggested favored fat storage) rather than fat storage per se.

The TGH is that humans have always been living in an environment characterized by sparse and unpredictable food supplies. Hence, it was argued that when food supplies failed it would have been those individuals who stored more fat would have had a greater chance of survival. This means that the genes favoring fat storage were positively selected—higher SP or higher LIP in a DIP model. As noted elsewhere, this is an intuitively attractive model [83], but it has almost no supportive evidence, and there is considerable evidence against it. Even Neel himself published a paper later in his career indicating the original idea was perhaps naïve [84]. Yet, despite this overwhelming evidence, it refuses to die [85], and the original paper has already been cited more than 2000 times.

The problems with the TGH have been elaborated elsewhere [9, 83, 86–90] but will be briefly summarized and added to here. The first issue is that there is no evidence to support the suggestion that we have always been inhabited a niche characterized by low and intermittent food supply. Indeed, in some scenarios, pre-agricultural hominids (e.g. *Homo erectus*) were an apex predator that was awash with abundant energy supplies from the vast African populations of large mega-herbivores such as elephants [91, 92]. Moreover, early hominids and early populations of *Homo sapiens* have been characterized as driving the extinction of these prey items because they killed many more than were needed for food—the overkill hypothesis [93, 94]. Overkill is mutually incompatible with the idea of perpetual food shortage. Moreover, once hominids discovered fire, it became possible to cook food items and this greatly increased the energy that could be obtained from plant sources [95]. By this scenario, food shortages probably only started to affect humans once elephants and other mega-herbivores [96] had been extirpated. This led ancestral humans to rely on smaller and smaller prey and an increasingly large diversity of plant and other food sources such as honey [92] until the agricultural revolution. Once humans developed agriculture 8–10k years ago, this did not stabilize the food supply and make it more predictable, but rather famine started to become a more-regular feature of human existence. Food security is higher in hunter–gatherers than subsistence agriculturalists [97]. Moreover, much larger historical populations became highly susceptible to crop failures and unpredictable weather events [98–100].

So, the idea of thrifty genes promoting survival through famines only makes sense in the period since we developed agriculture [100]. Although there are several examples of genetic selection acting over the period since animals were domesticated (e.g. in the lactase gene [101]), the problem with the famine-based selection acting only in the agricultural era is that 10k years is insufficient time for famines to act as a strong selective force on genetic variants influencing fat storage, because catastrophic famine events are relatively rare (every 150 years or so) [86]. More critically for the TGH, however, there is no evidence that the people who survive famines are any fatter than those who die. The reason for this is that famine seldom involves a complete failure of food supply where individuals are forced to rely exclusively on their fat reserves. Females who are on average fatter than males do survive famines better, but this bias is unlikely to be causally linked to their fatness [102]. The individuals who survive famines are not those who start the famine fattest, but the ones who can dominate the available food. This means that mortality is concentrated on young (aged <5 years) and older individuals (aged >40 years) [86]. Such mortality is unlikely to be biased toward leaner individuals in the very young and, in older individuals, is likely irrelevant because they have already passed on their genes [86, 87].

One particular scenario often used to support the “famine” and TGH is the fact that modern Pacific islanders show extreme levels of obesity. American Samoa, Nauru, and the Cook islands have populations where obesity (BMI > 30) exceeds 63% of the population (for comparison, the USA is currently at 35%). This led to a hypothetical selection scenario where mortality was high (speculated to possibly be up to 75%) in individuals crossing to these islands and heavily biased against lean individuals [103]. This was combined with the fact that once they arrived the islands had only poor food supplies, perpetuating the selection of those able to store the most fat. These ideas have been advanced largely with scant regard for the archeological and anthropological evidence regarding pacific island exploration [104]. In particular, the notion that groups of individuals climbed into boats to partake in perilous journeys of adventure to unknown remote destinations, during which most of them died of starvation, so that only the fat ones got off at the other end [105] is completely fanciful. The reality is that most colonization trips involved preliminary two-way voyages to explore potential destinations, followed by the departure of well-provisioned colonization parties who were traveling to known destinations with known resources to last them for a journey of known duration [104, 106]. This is combined with the fact that often the island destinations have abundant food resources such as fish and shellfish.

Nevertheless, Pacific islanders do appear to have enrichment of some alleles that predispose them to obesity. For example, Furusawa *et al*. [107] found an association between polymorphisms in the LEPR and obesity in pacific islanders. Minster *et al*. [108] found that a normally rare missense variant (rs373863828) in the *CREBRF* gene was strongly associated with BMI in the Samoan population, where it is relatively common (frequency = 0.259). Although they suggested that this allele had been subject to positive selection, establishing whether the enhanced allele frequency was a consequence of positive selection due to enhanced survival during famine events or founder effects followed by rapid population expansion is challenging. This finding was later replicated in other Polynesian populations [109] where it was shown to not only increase BMI but also *reduce* the risk of developing type 2 diabetes. More recent work suggests that this missense variant actually impacts BMI via an effect on fat-free mass rather than fat mass [110], thus explaining the paradoxical effects on BMI and type 2 diabetes risk, and making the possibility it is a “thrifty variant” related to fat storage as originally claimed remote.

If periodic unanticipated famines are not the key driver for fat storage, then another argument is that human body fatness evolved to tide us over more predictable periods of food shortage, in particular winter. This annual food shortage overcomes the issue that the number of selection events in periodic famines is too small to select for genes linked to fat storage, since winter is an annual event 150 times more common than a famine. The argument takes as its baseline the observation that generally winter in the northern hemisphere involves periods of harsh cold where energy expenditure for thermoregulation would likely be elevated and this is combined with a time period when food supply is reduced because of reductions in primary productivity. Moreover, there are numerous examples of wild animals that store large amounts of body fat to get them through this period of food shortage, including bats, bears, and ground squirrels. Indeed, this idea has been expanded to suggest that our predilection for foods that promote fattening is because these foods mimic the macronutrient composition of foods generally available in the fall and on which we would have supposedly gorged to deposit our winter fat store [111]. Again, this argument is developed largely devoid of any consideration of the actual facts. Studies of wild mammals generally show that energy expenditure is seldom elevated in winter despite it being substantially colder [112]. In fact, very often energy demands in winter are substantially reduced compared with summer, even in animals that do not hibernate, because of behavioral and metabolic adaptations [113,114]. Moreover, the species that deposit fat stores in the fall are most often species that also hibernate and hence have little opportunity to feed during the winter because they are torpid, necessitating them to deposit fat in advance. Perhaps, the only evidence in favor of this idea is that there is direct evidence in some species that fatter individuals do have a greater chance of over-winter survival [115]. Nevertheless, among species that do not hibernate, it is often observed that they store the largest fat reserves during summer rather than winter to support reproduction [116–118].

Two other facts mitigate against this hypothesis. The first is that modern humans do not show a strong seasonal cycle in fat storage and deposition [77,119] but see the study of Westerterp [120] which would be expected if we had evolved to store fat to survive winter. Second, *H. sapiens* only left Africa and invaded more temperate climes in the last 40,000 years [121]. Hence, “we” have only been exposed to this selection pressure for the last 1%–2% of hominid evolution, and this exposure has been restricted to the subpopulation of hominids that left Africa. For most of our evolution, we were living in a largely temperature aseasonal tropical environment. One might then expect if this was an important evolutionary factor that there would be large differences in fat storage between Caucasian- and African-derived populations when inhabiting a common environment—and while this is true, the direction of effect (greater obesity levels in those of African origin in the USA) 122 is opposite to that predicted. Seasonality may also occur in the tropics particularly in rainfall patterns and with regard to agriculturalists who generate crops that are harvested at one time of year. This may lead to seasonal gluts in the energy supply. In these cases, crops are often stored in reserves external to the body that allow the seasonal glut in supply to be evened out across the year—in common with many animals that also store energy outside their bodies to survive winter (e.g. chipmunks, *Tamias striatus* [123]; North American red squirrels, *Tamiasciurus hudsonicus* [124]; and American pika, *Ochotona princeps* [125]) or support lactation [126].

While catastrophic failure of the food supply or seasonal declines in food is unlikely drivers of fat storage, it is still possible that fat evolved as a buffer against food shortage. This is because at the individual level a person may encounter situations that affect their ability to feed, despite there being abundant food resources available for everyone else. The main factor having this effect is infection with disease [90]. Disease often leads to suppression of hunger called “disease-induced anorexia.” This effect is probably adaptive. A person who is sick may not be effective at gathering food or hunting and may hasten their own demise if they do so, because they burn through their energy faster. Moreover, if debilitated by illness, they may themselves become prey to other animals. Hence, fat may be a buffer to protect against such periods of illness. Modern-day humans have these same physiological responses to infection, and there is lots of evidence that individuals with higher BMI can better survive infectious disease [127–129].

This argument may seem surprising in the light of recent evidence regarding infection with SARS-COV2 leading to COVID-19 and elevated risk of mortality in the obese [130]. Aged individuals and individuals with comorbidities, including obesity, diabetes mellitus, and hypertension, have significantly higher risks of hospitalization and death from COVID-19 infection than healthy individuals [131]. Older adults (>70 years) and individuals with obesity, type 1 or type 2 diabetes, hypertension, or atherosclerosis are all linked to a heightened inflammatory state associated with severe COVID-19 complications [132–140]. In addition, elevated fasting blood glucose is an independent risk factor for fatality in COVID-19 patients not previously diagnosed as having diabetes mellitus [141]. Men have over double the death rate of women [131, 135, 139]. Aging, chronic diseases, and male sex are all linked to an increase in the levels of systemic inflammation and endothelial dysfunction, which could explain a potential common pathway between these factors and COVID-19. Physiological changes caused by metabolic syndrome and type 2 diabetes mellitus are likely to synergize with COVID-19. Obesity is known to cause inflammation and insulin resistance in the vasculature and non-vascular tissues involved in glucose metabolism [142]. Adults with obesity and/or diabetes are no more likely to contract COVID-19 than people without obesity or diabetes, but they could be twice as likely to die from COVID-19 complications [143]. Although these studies clearly delineate a negative impact of high BMI levels (BMI > 40) on COVID-19 outcomes, the data on risk in individuals with lower levels of obesity are compromised by the way the reference group is defined—often by pooling together all the individuals without obesity (BMI < 30) and comparing those with individuals with obesity (BMI > 30). This pools heterogenous groups with lower BMI and obscures any potential impact of elevated risk in the very lean. In the few studies where the reference group can be disambiguated, there is a clearly increased mortality risk from COVID-19 among the very lean [139, 144], consistent with the idea that the LIP may have evolved as a buffer against infectious diseases. It has been noted that humans store considerably more fat than the great apes [145], and this difference is potentially a consequence of the greater risk of contracting infectious diseases in humans because of their greater sociality and living in larger groups.

Another potential use of stored body fat is in anticipation of elevated energy demands, rather than energy shortfalls. For example, fat is stored by migrating birds prior to migration [146]. Many animals also deposit fat stores in anticipation of reproduction, which is also a period of high energy requirements. Indeed, there is a distinction made between animals that predominantly rely on such reserves (like pinnipeds) called “capital breeders” and animals that predominantly utilize elevated intake to fuel reproduction such as mice [147] called “income breeders.” There is substantial evidence to suggest a close link between fat reserves and reproductive function in females which is mediated via circulating leptin levels. The ob/ob mouse, for example, is infertile, but reproductive function can be regained by exogenous provision of leptin [148]. Females with low levels of body fat become amenorrheic [149, 150]. It is noteworthy that the levels of body fat in nonobese individuals of the same BMI are much higher in females than males pointing to support of reproduction as an important driver of these baseline levels of adiposity.

### Why is there an upper limit on fat storage?

On 11 March 2011 at 14.46 Japan standard time, there was a massive magnitude 9.0 earthquake undersea 72 km east of the Oshika Peninsula. It triggered an enormous tsunami that hits the eastern Japanese coast several hours later breaching tsunami defenses in many coastal towns. A valid question to ask is why the defensive walls had not been built higher? The answer is that walls cost money. The Kamaishi seawall, 1.2-km long and 5-m high, costs 1.5bn US$ to construct, and Japan has 22,000 km of similar Pacific coastline to protect against tsunami events. Although you can always build a higher wall, the costs of doing so increase disproportionately as the wall gets higher, but the incremental protection they give gets smaller and smaller, because the probability of a more extreme tsunami gets progressively lower. It is the same with storing fat. Storing fat costs energy. That is because of two reasons. First, you have to carry it around and the heavier the fat store you carry the greater the costs of locomotion [151]. Second, fat is always stored with a certain amount of lean tissue, and hence there is an increase in basal metabolic rate as well. Therefore, people with greater fat stores have greater daily energy demands [152, 153] that have to be fueled by greater food intake. However, the additional protection provided by storing extra energy against a catastrophic shortfall in supply (e.g. during disease-linked anorexia) gets progressively lower because the probability of events lasting longer is reduced. Another point is that while humans can anticipate hypothetical events that have not happened and act accordingly (e.g. anticipating a future tsunami that is much larger than the 2011 one), evolution cannot do so. Selection can only act on events that have actually happened. The rarer those events are the lower the selection pressure.

The above is an argument for why there would be little selective pressure to store considerably more fat than the SP or the LIP. A symmetrical SP or DIP model, however, implies not only the absence of a selective pressure pushing up fat levels but also the existence of some counter-balancing pressure capping their increase. This implies that storing fat above a certain level is disadvantageous (rather than insufficiently advantageous). There is abundant evidence, suggesting that this counter-balancing pressure is the risk of predation.

The idea that fatness is a trade-off between risks of starvation, on the one side, and risks of predation, on the other side, dates back to the 1980s [154–156]. Although the starvation risk side of this balance, as we have discussed above seems unlikely, unless linked to disease risk, the predation side of the argument seems much better founded. There is much evidence supporting the idea that animals exposed to greater predation risk in the wild [157–159] and in experimental laboratory settings [160–162] reduce their levels of stored fat. Greater fat stores may be linked to a greater risk of predation either because storing more fat reduces maneuverability, and hence the ability to escape predator attacks, or because fatter individuals need to feed more to sustain their greater weight [152] and hence are more exposed to predators because of the longer time spent foraging. The molecular mechanisms that underpin the predation effect have been partially elucidated [163].

### Set-point or dual-intervention points?

Speakman [90,164] presented two mathematical models indicating why evolution might favor a DIP rather than an SP. These arguments were based firstly around the fact that the effects of body fat storage on mortality relating to starvation and predation would need to be unrealistically large to create a tight regulation system around a single point. When the mortality is less sensitive to body fatness and, in a more realistic range of values, the effects of declining risk of starvation and increasing risk of predation tend to cancel out over a quite wide range of body fatness. This would make the evolution of a DIP more likely [90]. The second argument revolves around the variation in the link between mortality and starvation/predation, which makes a selection on a single fixed SP unlikely [164]. Other models are available. Higginson *et al*. [165], for example, considered that the LIP and UIP might be linked and hence move together in relation to predation and starvation risks. However, this seems to be due to the assumptions built into the initial model, as independent variation of the UIP and LIP is certainly possible [164]. Overall, these models favor DIPs evolving rather than SP-based systems. Tam *et al*. [166] explored the responses of mice to perturbations of energy balance and concluded empirically that they did not have an SP control system for adiposity. In contrast, Hall and Guo [167] suggested that an SP was commensurate with current data in humans. In both cases, though a DIP was not explicitly considered.

## Question 3: What has gone wrong? If we evolved such a DIP control system, why is there an obesity epidemic?

It is important, at the outset, to note three things regarding evolutionary arguments about obesity and the obesity epidemic. The first point is that any adaptive argument about why we store *some* fat, for example, to survive periods of disease-related anorexia or to support reproduction, is not on their own sufficient to explain why some people get obese. The reason is that an adaptive argument needs to be quantitatively appropriate. The Hadza hunter-gatherers store enough fat to last 3–6 weeks without any food for 3 weeks in males and 6 weeks in females [168]. That is probably enough time to get an individual through a period of disease-induced anorexia for most diseases and enough to provide additional support for reproduction in females. In contrast, many people in modern society store enough fat to last them for more than a year with no food intake. We know that because extremely obese people have been entered into therapeutic fasting programs and fed no food for up to 382 days and they survive [169–171]. There is no known disease, or period of seasonal food shortage, for which such fat storage would be necessary. Hence, if the argument is made that obesity is adaptive, then the thing it is protecting against needs to be commensurate with the amount of fat stored. No such arguments have yet been advanced. However, this could conceivably be a reproductive investment that in human females may last several years via extended lactation [172].

The second point is that any argument that claims that obesity is adaptive (i.e. advantageous), e.g. the TGH, has to be able to explain why many people in modern society did not inherit the genetic variants disposing to obesity. Even in the USA after 50 years of exposure to an obesogenic environment, less than 40% of people are obese. Therefore, if one makes the argument that we store obesity levels of fat to support extended lactation, we need to understand why many people did not inherit the genetic variants that make that possible, if it is really advantageous. The third point is that our genes probably did not change significantly since the 1950s. It is possible for some enrichment of variants linked to obesity due to assortative mating [173] but that is not sufficient to explain the rise in obesity levels [174]. Hence, the modern obesity epidemic is a gene-by-environment interaction. Something in our evolutionary history gave “some” people the genetic propensity to store excess body fat. When embedded in modern society that genetic predisposition is expressed. Our concern here is in the factors that created that evolutionary pre-disposition rather than the modern environmental drivers. Saying people get fat because of the modern food environment is probably correct as far as the environmental side of the argument goes but is not an evolutionary explanation for the genetic predisposition.

Another potential factor that may have been important in driving up levels of obesity toward our UIPs is fetal programming. There is now abundant evidence that the period of early life when our brain circuits are developing may have a profound effect on later susceptibility to obesity. The first indications that this might be important came from the pioneering work of Barker and Hales who linked later life disease outcomes to birth weight. As the birth weight in part reflects the nutrient supply during gestation, these associations suggested that adverse events in gestation might program offspring to respond in later life [175]. This is often suggested to be “adaptive” in the sense that, if the fetus experiences energy shortage during development, it is programmed to become more obese as a preparation for energy shortage events later in life. Such adaptive interpretations, however, seem unlikely for a long-lived species such as humans because the correlation between an energy shortfall during gestation and the probability of encountering such conditions later in life must be extremely small. Nevertheless, despite the lack of evidence that it is adaptive, programming is a well-established phenomenon, and there are many mouse studies providing mechanistic insights into how it develops [176]. Achieved body weight in mice on variable fat diets is more influenced by the diet the mouse’s mother ate than what they eat themselves [177]. This effect could be contributing to the public health crisis we are now seeing. Put another way “you are what your mother ate.”

Obesity (BMI) has a large genetic component. Yet, the results of Genome Wide Association Studies (GWAS) indicate that the genetic variants that cause this variation in most individuals have only relatively minor effects on fatness. There are also monogenic forms of obesity where the impacts of genetic mutations have profound effects, but such cases are relatively rare, and often the result of consanguineous mating. Obesity in the vast majority of cases is a result of mutations in many genes that each has a minor effect. At the moment, there are at least 900 known genetic variants that contribute. These polymorphisms are by definition not fixed in the population. We all carry a unique complement of genes that govern how fat we may become and the variation between individuals is enormous. How is that compatible with the model proposed above of a DIP system limiting how fat we become? Given that it is thought the UIP of the DIP system evolved as a response to predation (see references above), then one interpretation is that this variation stems from something that happened in our past with respect to predation risk [9, 83, 90].

Three to four million years ago, the earliest representatives of our hominin lineage were the australopithecine and paranthropine primates. Compared with modern humans, these were small (*ca.* 25–35 kg) primates living either solitarily or in small groups. The area of Africa where they lived was populated by a large array of predators [90]. Our hominin ancestors were probably under tremendous predation pressure, and it is hard to imagine they did not have a system limiting their fat storage to reduce predation risk. Two million years ago, however, we had evolved into early Homo species like *H. erectus*. These species were much larger (55–66 kg), lived in highly social groups, had relatively large brains, developed tools and weapons, and discovered fire. In combination, it is suggested that these changes moved hominids up the trophic scale [92]. We started eating mostly plants [178], but by the time we were *H. erectus*, we were a top predator eating mostly large animals. Whether one accepts this interpretation or not, something that happened during this time is that the large populations of other predators become largely extinct. This could have been due to competition with early *Homo* species, due to direct killing by *Homo* species, or due to an entirely unrelated event like climate change. Whatever the cause the extinction of these animals combined with the ability of *Homo* species to defend themselves against the ones that survived probably massively reduced the risk of predation.

Under this scenario, the system controlling the UIP would still be in place but increasingly redundant. There would be no adaptive selective force to remove the limit but equally no selective pressure to remove mutations that altered it. For two million years, the system would have slowly fallen apart. Some people would be fortunate and acquire relatively few mutations, or opposing mutations, but others would be less fortunate and acquire mutations that would greatly elevate the point at which their system would kick into action to limit weight gain [9, 83]. Embed that into modern society with multiple environmental drivers to overconsume energy and the result is that some people put on enormous amounts of body fat, while others remain stubbornly lean despite all the obesogenic drivers (Fig. 2b). In this model, the genetic component of obesity is a nonadaptive consequence of the removal of predation risk deep in our evolutionary history, combined with random mutations in the UIP control system. Because these mutations are not adaptive but drift in evolutionary time, this idea has been called the “drifty” gene hypothesis [83].

**Figure 3:**
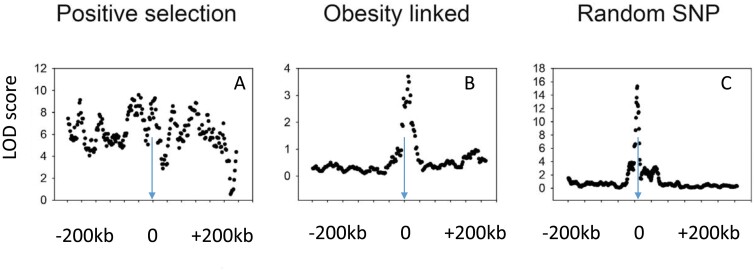
Three patterns of linkage disequilibrium (Logarithm of the odds scores) for single-nucleotide polymorphisms (SNPs) surrounding (200 kb upstream and downstream) three example target SNPs. The pattern, in (A), is an SNP (rs4988235) adjacent to the lactase gene that shows a significant association between the target and other SNPs over a wide surrounding distance. This is indicative of a selective sweep reflecting strong positive selection in this region of the genome. The pattern, in B, for a typical obesity-related SNP (rs1499209) by contrast shows a sharp peak around the target indicative of no strong selection and similar to the pattern, in C, for a randomly selected SNP (rs1420258) (data from the International HapMap Consortium: presented in [198])

Testing between the nonadaptive “drifty gene” hypothesis and adaptive interpretations such as the “thrifty gene” hypothesis based on famine events since the dawn of the agricultural revolution can be performed by looking for signatures of selection. There have been several previous searches for such signatures. Ayub *et al*. [179] searched for such signatures at 65 loci in 2 genes linked to type 2 diabetes, and Koh *et al*. [180] searched in both obesity and T2D gene variants identified by GWAS in East Asia and also found nothing. Southam *et al*. [181] looked for selection signatures in variants of 11 obesity-related genes and did not find any indication of selection. Finally, Wang and Speakman [182] investigated variants in 112 genes from GWAS of obesity. They found some limited evidence of positive selection in 5 genes, but this number was not different from a random sample of 112 variants in other non-obesity-related genes. Plus some of these selected alleles favored leanness rather than fat storage. Overall, there is scant evidence that the genetic variants predisposing to obesity have undergone strong selection (Fig. 3). This strongly favors the “drifty gene” interpretation over adaptive interpretations including the TGH.

There are various other indicators that obesity is not an advantageous trait that was positively selected, for example, to allow us to survive famines. Probably, the most comprehensively investigated of these is the impact of obesity on physical attractiveness. If obesity was a trait positively linked to survival that had been the product of adaptive evolution, then we would expect it to become a secondary sexual characteristic. Individuals who carry large fat stores would be providing an honest signal of their ability to survive energy shortfalls, and hence they should be seen as more attractive than leaner individuals who would be more likely to die. Yet, there are many studies that have looked at the links between body composition and physical attractiveness, and they universally find across all cultures that the most attractive female phenotype is thin—having a BMI of about 18–20 [183, 184]. This corresponds to the typical level of fatness in a female aged 16–20 years at peak fertility. In males, the situation is more complex because carrying large muscle mass increases both attractiveness and BMI, but increasing BMI due to elevated fatness is not attractive in males. Obesity is clearly not a secondary sexual attractant, and these data indirectly support the nonadaptive drifty gene idea.

## Implications

If there is a DIP system, as seems more likely to have evolved, then this implies in the brain that there must be two systems centered on the LIP and UIP, rather than a single SP. It seems likely that the LIP is at least in part based on low levels of leptin. When leptin levels fall, that indicates a decline in body fatness and results in coordinated neuroendocrine responses and a significant increase in food intake to rectify the situation. Increasing leptin levels does not evoke a similar reaction for two reasons. When leptin levels increase above the lower point, then the body mass is in the region where there is no physiological control. So, a response may not be necessarily expected. At higher levels, there may be no response because leptin is not the signal utilized at the UIP [185–190]. In this model, there is no need to invoke the concept of pathological leptin resistance to explain the responses to increasing leptin levels. The mechanisms explaining the asymmetric response to leptin at the LIP are still necessary to understand how the DIP works. The DIP model additionally suggests that there is likely some other regulatory mechanism, yet to be discovered, that governs fatness at the UIP [188–190]. The existence of such additional factors is indicated by close analysis of the results of parabiosis experiments [191]. Moreover, the fact that known gene variants identified by GWAS are dominated by centrally acting genes including the MC4Rs is consistent with the interpretation that these genes are something to do with the definition of the UIP [30].

An important question is how the UIP might work to cap the increase in body weight. One possibility is that once the UIP is reached compensatory changes in metabolism are affected. In this light, there is abundant evidence that the melanocortin system is linked to the regulation of BAT, and “browning” of WAT, which may form the basis of this effect. The first demonstration of an interaction between the melanocortin system and BAT thermogenesis was made in the late 1990s [192, 193]. Central administration of the synthetic melanocortin receptor antagonist, SHU9119, completely blocked leptin-induced increases in BAT *Ucp1* mRNA expression in rats [194]. Moreover, central administration of the MC4R agonist, MTII, increased BAT *Ucp1* mRNA expression—an effect that was blocked by surgical sympathetic denervation [195]. Transgenic mouse models further clarified the role of the leptin–melanocortin system in BAT thermogenesis. For example, compared with wild-type mice, mice lacking MC4R showed a smaller upregulation of BAT *Ucp1* in response to HFD or cold exposure (stressors that affect leptin production) [196]. However, whether leptin directly acts on the melanocortin circuit to regulate BAT activity needs further validation. Interestingly, MC3R and/or MC4R are required for exogenous leptin-induced increases in renal, but not BAT, sympathetic nerve activity in anesthetized rats [192].

Many MC4R-expressing neurons in the PVH are polysynaptically connected to BAT [197]; however, conditional re-expression of MC4R specifically in the PVH of otherwise MC4R-null mice did not restore impaired energy expenditure in these animals as assessed by indirect calorimetry [199]. Subsequent studies found that MC4R expression in sympathetic, but not parasympathetic, autonomic preganglionic neurons is important for regulating thermogenesis in mice [200]. Deletion of *Mc4r* in the sympathetic preganglionic neurons using choline acetyltransferase (ChAT)-Cre mice impaired HFD- and cold-induced thermogenesis in BAT and reduced the development of Beige Adipose Tissue (BeAT) [200]. These findings support the idea that cholinergic preganglionic neurons might be involved in the effects of leptin and melanocortin on BAT metabolism.

Although the specific MC4R-expressing neuronal populations that regulate BAT thermogenesis are unknown, recent work has suggested a role for ARC neurons. As mentioned, the ARC is a heterogeneous region of the hypothalamus in which melanocortin neurons with opposing functions reside in close proximity to one another [201, 202]. Overexpression of X-box binding protein 1 (XBP1), an important molecule in the endoplasmic reticulum stress response, in POMC neurons is sufficient to increase thermogenesis in BAT and browning of inguinal WAT [203]. Thus, XBP1 in POMC neurons may regulate the development of BeAT and thus contribute to improvements in whole-body metabolism. Interestingly, blocking the connexin 43 gap-junction system in WAT prevents the ability of neuronal POMC XBP1 to affect BeAT development, suggesting that the effects of the melanocortin system on BeAT metabolism require an intact adipocyte gap junction system [204]. Furthermore, insulin and leptin act together on POMC neurons to promote BeAT formation [205]. Deletion of the tyrosine-protein phosphatases, such as PTP1B and TCPTP, which regulate insulin signaling in POMC neurons prevents HFD-induced obesity by increasing BeAT development and energy expenditure [205]. Together, these studies indicate that POMC neurons are important for regulating BAT, WAT, and BeAT metabolism. Whether these systems are enabled as individuals exceed their UIP remains to be demonstrated.

The ob/ob mouse becomes extremely obese, as do humans with loss-of-function mutations in the leptin gene. If there is a DIP controlling fatness, then presumably this increasing fatness must, at some point, hit the UIP. Indeed, ob/ob mice do stop gaining weight and the point at which they do so is not the level of maximum possible fatness defined by, for example, physical constraints of the body. We know this in part because if the hormone adiponectin is deleted in ob/ob mice, the double knockout mice become even fatter than the single leptin-deficient mice [206]. However, when adiponectin is transgenically over-expressed in ob/ob mice, it results in increased body weight and adiposity, especially the subcutaneous fat depots [81]. An interesting question then is what causes the ob/ob mouse to stop gaining fat, because that may provide us with a clue about the UIP. This could perhaps be usefully explored by looking at the impacts of knocking out the leptin gene on different background mouse strains and then correlating the brain gene expression with the attained adiposity. Important to note, however, that if it turns out that fatness is not regulated by a DIP model, then searching for the molecular mechanism of that system is as fruitless as trying to understand pathological leptin resistance if there is no SP. Clearly, there is a need for studies that elucidate whether the system is regulated by an SP or DIPs.

Another implication of the DIP model is that it provides a potential explanation of the variation in response of individuals to interventions that aim to reduce body weight. Most studies of weight loss in response to calorie-restricted diets or exercise programs show a profound variation in the resultant weight loss. One interpretation of these outcomes is that they reflect variation in adherence to the intervention. That rests on the assumption that, if everyone had adhered to the program, then they would all have lost similar amounts of weight. However, King *et al*. [207] performed an exercise intervention program where people were supervised to do the required exercise, so adherence was not an issue. Surprisingly, they found the same pattern of variation in response. This suggests then that under an exercise intervention some people invoke compensatory responses to the elevated expenditure, which oppose the weight loss, while others do not and they lose significant amounts of fat and weight. Why such variability exists can be understood using the DIP model. That is, if most people at the start of the intervention are in the region between the regulation points, then they would have variable amounts of weight to lose before they hit their own LIP at which point they would invoke compensatory responses to prevent further loss. If this interpretation is correct, then all things being equal one would expect people who started out heavier to lose more as they would be more likely to be further from their LIP. Regardless, abundant evidence in human subjects has demonstrated that exercise training alone is not an effective weight-loss strategy. However, training does induce favorable changes in body composition and in several metabolic parameters. While physiological responses to exercise are established, especially in humans, the mechanisms underlying these effects are poorly understood. It is likely that exercise training affects the control mechanisms and brain pathways discussed above to mediate these effects.

## Conclusions

Body fatness appears to be regulated. How exactly it is regulated (SP versus DIP) and the evolutionary background to the evolution of this system remain uncertain. There is increasing consensus that low levels of adiposity elicit responses to elevate fatness mediated via a system dependent on leptin and melanocortin signaling. High levels of adiposity may also elicit responses probably not involving changes in leptin but potentially still involving the melanocortin system. Metabolic programming in early life dependent on the nutritional exposures of our parents may influence this system. Evolutionarily, it is important to distinguish reasons why we store some fat, from reasons why we store lots of fat in obesity. The former is probably an adaptive response that evolved to reduce the risk of starvation, particularly during periods of anorexia induced by illness. The latter is probably due to the maladaptive degeneration of a control system that evolved to limit fat accumulation because of the risk of predation. Once we developed tools, fire, and social behavior, we effectively reduced the risk of predation to extremely low levels and mutations in the control system constraining high levels of adiposity were not eliminated and accumulated in time—the “drifty gene hypothesis.” Future work needs to focus on establishing if we have an SP regulation system or a DIP system and the molecular nature of regulation system that limits the accumulation of body weight/fat.
